# Time-varying effects of local weather on behavior and probability of breeding deferral in two Arctic-nesting goose populations

**DOI:** 10.1007/s00442-022-05300-x

**Published:** 2022-12-28

**Authors:** Stephanie A. Cunningham, Toryn L. J. Schafer, Christopher K. Wikle, Jay A. VonBank, Bart M. Ballard, Lei Cao, Stuart Bearhop, Anthony D. Fox, Geoff M. Hilton, Alyn J. Walsh, Larry R. Griffin, Mitch D. Weegman

**Affiliations:** 1 School of Natural Resources, University of Missouri, Columbia, MO 65211 USA; 2 Department of Statistics, University of Missouri, Columbia, MO 65211 USA; 3 Department of Statistics, Texas A&M University, College Station, TX 77843 USA; 4 Caesar Kleberg Wildlife Research Institute, Texas A&M University-Kingsville, Kingsville, TX 78363 USA; 5 State Key Laboratory of Urban and Regional Ecology, Research Center for Eco-Environmental Sciences, Chinese Academy of Sciences, Beijing, 100085 China; 6 University of Chinese Academy of Sciences, Beijing, 100049 China; 7 Centre for Ecology and Conservation, College of Life and Environmental Sciences, University of Exeter, Cornwall Campus, Penryn, TR10 9EZ UK; 8 Department of Bioscience, Aarhus University, C.F. Møllers Allé 4-8, 8000 Aarhus C, Denmark; 9 Wildfowl & Wetlands Trust, Slimbridge, GL2 7BT Gloucester UK; 10 National Parks and Wildlife Service, Wexford Wildfowl Reserve, North Slob, Wexford Ireland; 11 ECO-LG Limited, Crooks House, Mabie, DG2 8EY Dumfries UK; 12 Department of Biology, University of Saskatchewan, Saskatoon, SK S7N 5E2 Canada; 13 Present Address: Department of Environmental Biology, State University of New York College of Environmental Science and Forestry, 1 Forestry Drive, Syracuse, NY 13210 USA

**Keywords:** Accelerometer, Ecological barrier, Energy expenditure, Reproduction, Stopover, Time-varying covariates

## Abstract

Arctic-nesting geese face energetic challenges during spring migration, including ecological barriers and weather conditions (e.g., precipitation and temperature), which in long-lived species can lead to a trade-off to defer reproduction in favor of greater survival. We used GPS location and acceleration data collected from 35 greater white-fronted geese of the North American midcontinent and Greenland populations at spring migration stopovers, and novel applications of Bayesian dynamic linear models to test daily effects of minimum temperature and precipitation on energy expenditure (i.e., overall dynamic body acceleration, ODBA) and proportion of time spent feeding (PTF), then examined the daily and additive importance of ODBA and PTF on probability of breeding deferral using stochastic antecedent models. We expected distinct responses in behavior and probability of breeding deferral between and within populations due to differences in stopover area availability. Time-varying coefficients of weather conditions were variable between ODBA and PTF, and often did not show consistent patterns among birds, indicating plasticity in how individuals respond to conditions. An increase in antecedent ODBA was associated with a slightly increased probability of deferral in midcontinent geese but not Greenland geese. Probability of deferral decreased with increased PTF in both populations. We did not detect any differentially important time periods. These results suggest either that movements and behavior throughout spring migration do not explain breeding deferral or that ecological linkages between bird decisions during spring and subsequent breeding deferral were different between populations and across migration but occurred at different time scales than those we examined.

**Supplementary Information:**

The online version contains supplementary material available at 10.1007/s00442-022-05300-x.

## Introduction

Long-lived species are expected to forego reproduction when conditions are such that expending energy in a reproductive attempt would reduce residual reproductive value by compromising survival or future reproductive success (Ankney and MacInnes 1978; Lindén and Møller 1989; Erikstad et al. 1998). While numerous studies have investigated how weather patterns influence migration flights in individuals, as well as overall impacts on populations (Shamoun-Baranes et al. 2017 and references therein), conditions at wintering or staging areas have also been linked to subsequent variation in demographic rates and life-history tradeoffs in birds (van Oudenhove et al. 2014; Dybala et al. 2013). Conditions driven by large-scale climate patterns are responsible for lower reproduction in years when environmental conditions are less favorable (Cubaynes et al. 2011; Cleasby et al. 2017). In addition to weather conditions, physical characteristics of migration routes can present challenges during migration. While birds undertaking an overall longer migration may be challenged by increased energy requirements, they generally have more flexibility to adjust their migration to conditions they encounter (Sorte and Fink 2017). Large water crossings such as oceans can diminish the predictability of conditions from one stopover location to the next and therefore reduce a bird’s ability to respond to changes in timing of the onset of spring, which can affect forage quality (Tombre et al. 2008) and can influence reproductive success (Lameris et al. 2018).

Arctic-nesting geese obtain resources for reproduction before and during migration, as well as after arrival to breeding areas (Gauthier et al. 2003; Drent et al. 2007). The stopover areas birds use to refuel and build fat and protein stores before continuing migration are of particular importance (Weber et al. 1998), as endogenous reserves explain variation in clutch size of Arctic-nesting geese (Alisauskas 2002; Inger et al. 2010) and affect reproductive success and breeding mortality (Ankney and MacInnes, 1978). Precipitation, e.g. snow, at staging areas can influence food availability (e.g., Webb et al. 2010; Haest et al. 2020) in addition to temperature affecting individual energy expenditure via increased thermoregulation (Wiersma and Piersma 1994; Bauer et al. 2008), with subsequent influence on reproductive performance (Inger et al. 2010; Harrison et al. 2011; van Oudenhove et al. 2014). Conversely, there is some evidence that migratory waterfowl can compensate for poor winter conditions during spring migration or when they arrive on breeding areas if conditions allow for better foraging opportunities (Steenweg et al. 2022).

**Fig. 1:**
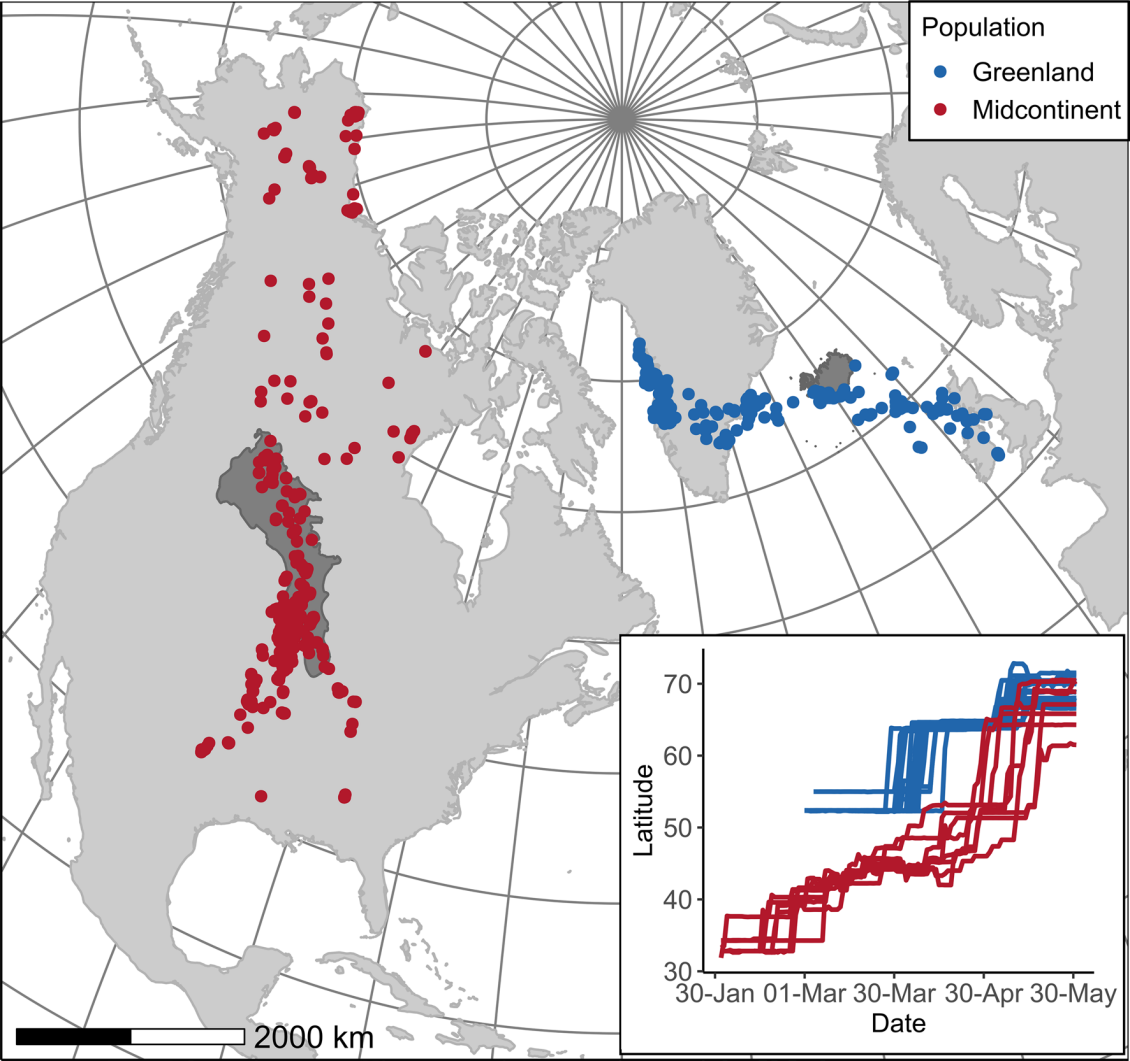
Migration locations of 10 North American midcontinent (red) and 25 Greenland (blue) greater white-fronted geese tracked via GPS across North America and northwest Europe in 2012–2013 (*n* = 15) and 2017–2018 (*n* = 20). Shaded areas (Prairie Pothole Region in North America and Iceland) indicate staging areas from which the last day in these regions was used to determine the end of the migration period for each bird. Inset shows latitudinal movements of migrating geese by date

Herein, we focused on two populations of greater white-fronted geese (*Anser albifrons*): the Greenland subspecies (*A. a. flavirostris*) and the North American midcontinent population (*A. a. frontalis*). These populations exhibit contrasting population trends; the population size of midcontinent geese has been stable or increasing in recent years (U.S. Fish and Wildlife Service 2020; R. Alisauskas, unpublished data) while the Greenland population has declined 39% since 1999 (Fox et al. 2020). Greenland geese cross the North Atlantic from wintering areas in Britain and Ireland to Icelandic staging areas, and to breeding areas in western Greenland, with relatively few sustained stops (Fox et al., 2003). In contrast, midcontinent geese migrate entirely over land from southern US wintering areas to breeding areas in the Canadian and Alaskan Arctic, and despite the large tracts of boreal forest in Canada, have opportunities for much more frequent, shorter stops to refuel (Ely et al. 2013; VonBank 2020; Fig. 1). Thus, geese within each population encounter distinct conditions and barrier-related stopover opportunities as they travel to the Arctic, which we anticipate might yield differences in the proportion of birds initiating a nesting attempt or choosing to defer.

We employed two novel applications of modeling approaches to investigate the daily and cumulative effects of weather conditions on goose behavior and how behavior relates to subsequent probability of deferring reproduction in white-fronted geese. We aimed to demonstrate the use of these methods in the context of understanding linkages among environmental conditions, behavior, and reproductive success, and in identifying critical time periods in the annual cycle of these geese. Our first objective was to assess the influence of fine scale (i.e., daily) temperature and precipitation patterns on daily energy expenditure (ODBA) and proportion of time spent feeding (PTF) during spring migration and the pre-laying period via dynamic linear models (e.g., Holmes et al. 2019; Laine 2020). The analysis of time-varying coefficients will allow us to identify potential bottlenecks in resource accumulation due to unfavorable conditions. We predicted that PTF and ODBA would be positively associated with minimum temperature because low temperatures were expected to increase energy expenditure due to increased energy demands of thermoregulation (Krams et al. 2010) and negatively associated with precipitation, especially in the case of snow, which may hinder foraging (Reed et al. 2004). We predicted that weather conditions would impact geese similarly within a population, with the potential for differences between birds attempting (or successful) and deferring (or failed).

Next, we used a method described by Ogle et al. (2015) that builds on the concept of ecological memory, which is the contribution of previous experiences or states to current or future responses (Padisák 1992), to determine the extent to which ODBA and PTF during spring migration and the pre-laying period explain variation in probability of deferring reproduction for birds in each population. We predicted that greater antecedent PTF would be associated with a lower probability of deferral, while greater antecedent ODBA would be a predictor of increased probability of deferral, as we expected that ODBA would also be reflective of individual patterns of space use, and we expected these patterns to be more prominent in midcontinent birds, because Greenland geese are much more constricted in potential stopover areas (i.e., confined to Iceland in the middle of the ocean), and midcontinent geese have the potential to move across vast landscapes to find a suitable staging area. We predicted that if the model revealed differences in daily energy expenditure and time feeding between birds that attempted and deferred nesting, that the difference would be most pronounced late in migration, for example if the geese arrived on breeding areas and were faced with snow cover, decreasing their ability to recoup energy stores (Lameris et al. 2018) and therefore decreasing the likelihood of attempting reproduction. Because the mechanism causing the population decline in Greenland white-fronted geese is not fully known, we hoped that the application of these methods would allow for identification of critical time points, if any exist, that could influence whether an individual attempts or defers reproduction in a given year.

## Materials and methods

### Study populations

The midcontinent white-fronted goose population is estimated at > 1.3 million birds (U.S. Fish and Wildlife Service 2020) while the Greenland white-fronted goose population consists of approximately 21,500 birds (Fox et al. 2020). Habitat in Arctic regions of Alaska, Canada and Greenland consists mainly of tundra, characterized by moss- and lichen-covered uplands with flood-prone grassy lowlands and sedge meadows (Ely and Raveling 1984; Fox and Stroud 1988). The distribution of these birds across foraging habitats within their respective wintering areas differs between populations. The largest wintering flocks of Greenland birds congregate on agricultural fields near Wexford, Ireland and Islay, southwest Scotland, while ~ 70 other flocks are relatively small and typically less than a few hundred birds and show high fidelity to small geographical areas and to grass swards (Wilson et al. 1991; Warren et al. 1992). During the winter, midcontinent geese are much more itinerate and spread across agricultural landscapes of the southern United States and Mexico (Anderson and Haukos 2003; Ely et al. 2013), and individuals often use multiple areas within a single winter with a greater preference for arable crop types than the Greenland birds (VonBank et al. 2021).

### Goose captures and tracking devices

Greenland geese were captured on wintering areas in Ireland (52° 22′N, 6° 23′W) in 2012, 2013, 2017, and 2018, autumn staging areas in Iceland (64° 33′N, 21° 45′W) in 2016 and 2017, and in Scotland (52° 0′N, 4° 2′ W) in 2012 and 2013. Midcontinent geese were captured on wintering areas across Texas (32° 54′N, 99° 53′W; 28° 53′N, 98° 52′W; 27° 20′N, 97° 46′W) in 2016, 2017 and 2018. Geese were captured by cannon or rocket netting on both continents, as well as modified leg-hold traps in Texas (King et al. 1998), and were fitted with collars or backpack devices bearing GPS and accelerometer (ACC) technologies. The latter measured change in velocity in three directions (i.e., movement; Shepard et al. 2008; Gómez Laich et al. 2011).

Forty-one Greenland geese were fitted with backpack devices attached with elastic shock cord (e-obs GmbH, Grünwald, Germany, 39 g including tag and harness), and also received an orange neck collar (17 g) with an alpha-numeric code and matching white leg ring. Thirty-one Greenland geese and 50 midcontinent geese were fitted with Cellular Tracking Technologies (CTT) neck collars (CTT LLC, Rio Grande, NJ, USA; model BT 3.0 [54 g] and BT 3.5 [45 g]), and 14 Greenland geese and 7 midcontinent geese were fitted with Ornitela neck collars (Vilnius, Lithuania, model OrniTrack-N38; 38 g). Different CTT models were deployed sequentially, and prior to Ornitela devices. Greenland CTT devices were mounted to orange collars, while the Ornitela and midcontinent CTT collars were brown or gray. Devices were deployed across several captures, which averaged 15.5 birds per capture and ranged from 1 to 50, with an average of 4.1 midcontinent collars deployed per catch and 3.1 Greenland collars deployed per catch. Geese were sexed via cloacal examination. In 2012 and 2013, only males received tracking devices, otherwise adult females were chosen (with one exception in midcontinent birds). Ideally, our analysis would have included only female geese, but we considered males as proxies for assessing incubation indirectly because long-term pair bonds are common in geese (Black 1996) and males are known to guard incubating females (i.e., we anticipate males are relatively stationary when guarding an incubating female compared to males not associated with an incubating female; Dittami et al. 1977; Madsen et al. 1989; Samelius and Alisauskas 2006). We attempted to fit only one individual of a pair or family group with a tracking device to maximize independence of data, given that white-fronted goose families migrate together (Weegman et al. 2016c). GPS fixes were recorded at 1 point per day (e-obs devices), every 2 h (Greenland CTT devices), every 30 min (all midcontinent), or every 15 min (Greenland Ornitela).

Twenty-five Greenland (from 2012, 2013 and 2018; 15 backpacks, all males; 10 neck collars, all female) and 10 midcontinent individuals (from 2017 and 2018, 1 male and 9 females) had sufficient data to be included in the analysis (i.e., > 75% of expected daily accelerometer bursts through June, and not more than three days without a location out of every 3 days through July). Only 1 year of tracking was included in analyses for all birds and was generally the year following initial capture. Twenty of 31 Greenland geese fitted with neck collar transmitters with uniquely identifiable codes in 2017 and 2018 were resighted alive during regular surveys of Greenland white-fronted geese (i.e., Fox et al. 2020) or targeted searches ≥ 1 year after initial capture, and an additional 6 were seen > 6 months but < 1 year after capture, though tags were not transmitting data at these times. Based on estimated resight rates of Greenland white-fronted geese of approximately 0.86 at Wexford and 0.60 elsewhere throughout their range (Weegman et al. 2016a), we assumed low sample sizes were due to transmitter failure rather than collar-induced mortality. We were unsuccessful in relocating midcontinent geese due to an extensive wintering range, large flock sizes, and cryptic collar color.

### Processing and classification of ACC data

ACC data were recorded at 10 (CTT and Ornitela units) or 10.5 Hz (e-obs units) for a duration of 3 s, yielding ~ 30 points per axis, every 6 min. Prior to classification, we calibrated all devices according to manufacturer specifications. We used two CTT (BT 3.0 and BT 3.5), and six Ornitela units to calibrate devices according to manufacturer-specific specifications, to ensure a consistent baseline across units for converting ACC data from millivolts to gravitational acceleration (*g*).

Classification of data from e-obs devices is described in Weegman et al. (2017a). For collared geese, we filmed birds for behavioral classification between 1 day and 6 months post-tagging, collecting 54 h of video footage of wild Greenland white-fronted geese in Iceland and Ireland, encompassing nine CTT and nine Ornitela units, with filming occurring over 32 different days. We obtained 65.5 h of footage from two captive birds at Texas A&M-Kingsville, Texas, USA, which we rotated through three collars: Ornitela unit N38, CTT BT 3.0, and CTT BT 3.5. To increase the likelihood of capturing acceleration bursts on film, we increased the rate of ACC collection in two CTT devices deployed in Iceland from every 6 min to every 2 min for five days and collected approximately 6 h of footage from these birds, and ACC duty cycles for devices on captive birds were increased to every minute.

We documented goose behavior using the ‘JWatcher’ program (Blumstein et al. 2006), classifying behaviors as feeding, stationary, and walking, though we later combined feeding and walking, as geese do not regularly walk long distances during migration unless feeding (Weegman et al. 2017a), and to maintain consistency with e-obs units. Flight bursts were obtained from observed migration periods, based on GPS tracks for all device types, and stationary bursts were taken from video observations but supplemented with periods of overnight roosting for Ornitela and CTT units (Weegman et al. 2017a). All flight and stationary bursts based on GPS behavior were visually checked to ensure conformity with known ACC traces for each behavior (i.e., either extreme oscillation or stable line). Undoubtedly, geese exhibit more than three behaviors, but we assumed that maintenance behaviors such as preening would not be captured frequently enough by accelerometers to be classified, as Fox and Ridgill (1985) observed preening comprising < 5% of daily activity in geese.

We compared 37 min of video classifications between observers (SAC and JAV) to determine inter-observer reliability (Kaufman and Rosenthal 2009) and accepted that observers were classifying behaviors equally because > 95% of the video was assigned the same behavior. ACC bursts were extracted and assigned a behavior according to video time. Each burst was plotted and visually checked to ensure only 1 behavior was present during the 3-s burst, and the signature appeared reasonable for the behavior (e.g., bursts that were labeled ‘feeding’ but appeared as a straight line were removed, *n* = 89 bursts removed), as there may have been error introduced by reaction time while videos were being scored. We identified 797 flight bursts, 106 feeding bursts, 892 stationary bursts and 75 walking bursts from Ornitela units and 569 flight bursts, 90 feeding bursts, 1381 stationary bursts and 199 walking bursts from CTT units. Due to variation in number of bursts per behavior, 150 bursts of each behavior were randomly selected to be included in the tag-specific training sets so as not to artificially inflate overall accuracy. Because they were housed in a planted wheat field (i.e., with considerable bare dirt between rows of wheat), captive birds did not display feeding behavior representative of wild grazing, so all feeding bursts came from wild Greenland white-fronted geese.

We calculated a total of 37 summary measures to describe the acceleration behavior in each burst, based on metrics used in the AcceleRater web tool (Resheff et al. 2014). We tested five machine learning algorithms for behavior classification: K-nearest neighbors, classification and regression trees, random forest, linear discriminant analysis and support vector machines (Nathan et al. 2012). We split training data into 70% training and 30% test sets to test each of the five methods (e.g., Glass et al. 2020). We calculated the mean overall accuracy for each model from tenfold cross validation to select the best model (Nishizawa et al., 2013; Olden et al. 2008). Random forest and support vector machine algorithms both exceeded 95% overall accuracy. We selected the random forest algorithm to classify data from all tags, as this algorithm has been used to successfully classify behaviors from a variety of taxa (e.g., Fehlmann et al. 2017; Lush et al. 2016; Pagano et al. 2017; Tatler et al. 2018). Tag- and behavior-specific accuracy and performance metrics are shown in Table S1.

We used overall dynamic body acceleration (ODBA) as a proxy for energy expenditure from ACC data (Wilson et al. 2019). To increase consistency between devices, we used quantile mapping, a technique common in climate modeling for correcting bias (Piani et al., 2010; Reiter et al. 2018) using the package ‘qmap’ version 1.0–4 (Gudmundsson et al. 2012). Due to manufacturer settings, Ornitela ACC data were bounded, meaning that recorded values were forced between a minimum and maximum (i.e., − 2048 to 2047 mV). Therefore, we opted to stretch Ornitela and e-obs values to match CTT. We visually assessed the plots of the empirical cumulative density function of the CTT, Ornitela, and transformed Ornitela data and selected the empirical quantiles over smoothing splines as the most appropriate mapping function.

### Defining migration period and reproductive deferral

We considered the spring migration period to start no earlier than 14 days prior to wintering area departure of each GPS-tracked goose to incorporate preparations for departure; however, some geese were tagged < 14 days prior to departure from wintering areas (*n* = 5 for Greenland, *n* = 2 for midcontinent). We defined the end of the spring migration period for each goose as the end of the 14-day period after departure of the last major staging area defined in the literature (Prairie Pothole Region spanning Alberta to Manitoba and South Dakota for midcontinent geese, and west Iceland for Greenland geese; Fig. 1; Ely et al. 2013; Fox et al. 2014; Weegman et al. 2017b), because geese often stage in the Arctic or sub-Arctic prior to nest site selection (Fox and Bergersen 2005). Geese use these staging areas consistently and in large numbers to rebuild nutrient stores, generally for > 1 week just before moving to breeding areas (Fox et al. 2002; Anderson and Haukos 2003; Hübner 2006).

We classified geese as having attempted or deferred reproduction based on retrospective analysis of patterns in GPS and ACC data, following the methods described in Schreven et al. (2021), which can identify incubation events as short as 3 days and used median daily ODBA and standard deviation in latitudinal movements between 1 May and 31 July. Two midcontinent geese failed to transmit ACC data after the first week of June, so we followed the procedures for identifying incubation from only the GPS signals, which persisted through July. Reproductive outcomes (i.e., success or failure) of 15 male Greenland geese with backpack devices (2012–2013) were confirmed by resighting marked individuals associating (or not) with young on wintering areas (i.e., 5–8 months post-hatch; Weegman 2014; Weegman et al. 2017a).

### Weather covariates

GPS points from neck collars were thinned to one per day in the late afternoon, at approximately 1600 h local time (i.e., mean deviation from 1600 h was 39 min), to match frequency of backpack devices, using the package ‘adehabitatLT’ version 0.3.23 (Calenge 2006; Calenge et al. 2009). We interpolated missing GPS coordinates during spring migration (*n* = 35 across 13 individuals with e-obs backpack devices; Supplementary File 1: Figure S1) using the ‘move’ package version 3.2.0 (Kranstauber et al. 2019). The maximum number of consecutive missing locations was ≤ 3 days, so we expected that these missing locations would not negatively impact results, as the analyses were predominately based on fine-scale ACC data, and weather patterns are likely large enough to account for small imprecision in interpolated locations.

Minimum temperature (°C) data were extracted for each once-daily GPS goose location from the National Centers for Environmental Prediction (NCEP)/Department of Energy Reanalysis II data set (2.5 × 2.5-degree spatial resolution; Kanamitsu et al. 2002) using the package ‘RNCEP’ version 1.0.1 (Kemp et al. 2012) in Program R version 4.0.2 (R Core Team 2020). The ‘RNCEP’ package provided four interpolated values (corresponding to approximately 0400, 1000, 1600 and 2200 h local time) at each location, which were averaged to obtain a daily value. We downloaded daily precipitation data from the Global Precipitation Climatology Project (GPCP) Version 1.3 (1-degree spatial resolution; Huffman et al. 2001; Adler et al. 2017) and extracted values using the R package ‘raster’ (Hijmans 2022). Precipitation data were missing across much of the raster for 6 April 2013, and we interpolated these values by averaging the values of the day before and day after. Daily temperature and precipitation were not strongly correlated (*r* = 0.15, 95% CI 0.12, 0.18).

### Statistical analyses

We developed Bayesian hierarchical models and implemented them in JAGS using the package ‘jagsUI’ version 1.5.0 (Plummer 2003; Kellner 2018) in Program R version 4.0.2 (R Core Team 2020). Convergence was confirmed via the Gelman-Rubin statistic (Brooks and Gelman 1998) and visual inspection of traceplots. Continuous variables were standardized to have a mean of 0 and standard deviation of 1. We log-transformed ODBA to meet normality assumptions.

#### Impact of daily conditions on ODBA and PTF

We modeled the relationship between lnODBA and weather conditions (minimum temperature and precipitation) using a dynamic linear model. Dynamic linear models are a form of state space models for time series with coefficients that evolve over time according to a temporal process such as a random walk autoregression model. The daily effects of each weather covariate on lnODBA were estimated for each individual (i.e., one model per goose yielded estimates for each day of that bird’s migration). A linear regression model with dynamic coefficients was used to model daily effects of weather covariates on median daily lnODBA. For each individual *i*, the model was specified as:

$${\mathrm{ODBA}}_{it}\sim \mathrm{Normal}({\mu }_{it},1/{\tau }_{i})$$

$${\mu }_{it}={\beta }_{0,i}+{\beta }_{1,it}\times {PRCP}_{it}+{\beta }_{2,it}\times {\mathrm{MTEMP}}_{it}$$

where PRCP_*t*_ and MTEMP_*t*_ were precipitation and minimum temperature, respectively, for day *t*. ${\beta }_{0,i}$ represented the intercept and had a vague normal prior with mean = 0 and variance = 100. ${\beta }_{1,it}$ and ${\beta }_{2,it}$ were the slope parameters for the effects of covariates on day *t*. The priors for the effect on the first day, ${\beta }_{1,i1}$ and ${\beta }_{2,i1}$ were normal with mean = 0 and variance = 100. The expected value of each day for each individual was represented by ${\mu }_{it}$, and the observation variance was represented by $1/{\tau }_{i}$. We used a gamma prior with shape = 0.1 and rate = 1 for the observation precision ${\tau }_{i}$. The dynamic evolution of the regression coefficients ${\beta }_{1,it}$ and ${\beta }_{2,it}$ was modeled independently as:

$${\beta }_{k,it}=\mathrm{Normal}({\psi }_{k}\times {\beta }_{k,i\left(t-1\right)}, 1/ {\eta }_{k,i})$$

where we assumed a random walk—and therefore imposing strong autocorrelation between the estimates—by fixing ${\psi }_{k}$ to 1 for all *k,* and where ${\eta }_{k,i}$ was process precision for covariate *k*, which had a gamma prior distribution with shape = 0.1 and rate = 1. We sampled three Markov Chain Monte Carlo (MCMC) chains, each with 120,000 iterations and a burn-in of 80,000, yielding 120,000 posterior samples.

We used the same approach for the effects of weather on proportion of time feeding, but replaced the linear model with a binomial generalized linear model, with the response consisting of the number of bursts classified as feeding and the total number of bursts such that:

$${\mathrm{PTF}}_{it}\sim \mathrm{Binomial}({y}_{it},{n}_{it})$$

$$\mathrm{logit}({y}_{it})={\beta }_{0,i}+{\beta }_{1,it}\times {\mathrm{PRCP}}_{it}+{\beta }_{2,it}\times {\mathrm{MTEMP}}_{it}$$

The priors for the effect on the first day, ${\beta }_{1,i1}$ and ${\beta }_{2,i1}$ were normal with mean = 0 and variance = 2.25. All other aspects of the model were the same as for lnODBA. We chose a smaller prior variance for the models using the logit link. When the covariates are standardized, it is unlikely to observe logistic regression coefficients on the order of 5, because that corresponds to a change in the probability of 0.5 (i.e., a probability of 0.01–0.5 for a coefficient of 5; Gelman et al 2008). Therefore, we chose the variance such that the prior puts small mass on coefficient values greater than 5 in absolute value.

#### Influence of ODBA and PTF on probability of reproductive deferral

We used a stochastic antecedent model (Ogle et al. 2015) to quantify the extent to which daily and cumulative lnODBA and PTF during spring migration explained variation in the probability of an individual deferring reproduction. These stochastic antecedent models include an antecedent variable as a cumulative measure of covariate values (i.e., lnODBA or PTF) weighted by the importance of each day (Ogle et al. 2015). If the antecedent variable explained substantial variation in the probability of breeding deferral, then a larger weight for one day would indicate that specific day significantly affected probability of deferral more than other days during spring migration. This may reveal time-lags in effects (e.g., if lnODBA or PTF during staging was more important than lnODBA or PTF on breeding areas in the days leading up to incubation; Ogle et al. 2015). We used a logistic regression for the likelihood of breeding deferral given the antecedent effects over a span of 54 days, which was the shortest-duration migration. Due to the calculation of daily weights, we were unable to include differing-length migrations, and instead used the last 54 days of migration for each goose, though days did not necessarily match calendar dates. We included only geese with neck collars (*n* = 20) to allow for an unbiased comparison between populations. The approach can be mathematically described as:

$${Y}_{i}\sim \mathrm{Bernoulli}({p}_{i})$$

$$\mathrm{logit}\left({p}_{i}\right)=\alpha +{\beta }_{1}\times {\mathrm{ant}X}_{i}+{\beta }_{2}\times {\mathrm{pop}}_{i}+{\beta }_{3}\times {\mathrm{ant}X}_{i}\times {\mathrm{pop}}_{i}$$

where *Y*_*i*_ was the binary response variable (1 for defer; 0 for attempt) for individual *i, β*_*k*_ represented slope parameters for *k* = 1, 2, 3, which were the realized effects of the antecedent variable, Greenland or midcontinent population, and the interaction between these, on probability of deferral. The antecedent variable for individual *i* is noted as *antX*_*i*_ and population of each individual is represented by *pop*_*i*_ (midcontinent = 0, Greenland = 1). A vague normal prior was used for *β*_*k*_ with mean = 0 and variance = 2.5. Following Ogle et al. (2015), antecedent variables were calculated as:

$${\mathrm{ant}X}_{i}=\mathop{\sum }\limits_{t=1}^{D}{X}_{i}(t)\times {w}_{X}(t)$$

where *D* indicated the duration of migration period in days, *X*_*i*_(*t*) was the daily value of PTF or lnODBA for individual *i*, and *w*_*X*_(*t*) was the daily weight. A Dirichlet prior was used for weights (specified via the gamma distribution in JAGS with rate and shape = 1). MCMC chains each had 5000 iterations and burn-in 2500 samples, yielding 7500 total posterior samples over three chains. The daily and cumulative weights estimated from the stochastic antecedent models were examined to determine temporal variation in importance of lnODBA and PTF.

## Results

Migration duration varied substantially between populations. While Greenland geese did not depart winter quarters in Ireland and Scotland until early April, midcontinent birds began moving north as early as late February or early March (Fig. 1, inset). Based on movement and lnODBA characteristics of collared birds, 3 out of 10 Greenland birds with collars deferred nesting, and 4 of 10 midcontinent birds deferred (Supplementary File 1: Table S1). Two out of 15 Greenland geese with backpack devices were confirmed as having successfully raised young. Median daily lnODBA ranged from − 4.4 to 0.35 for Greenland geese with collars, − 4.5 to 0.51 for Greenland geese with backpacks, and − 4.3 to 0.44 for midcontinent birds. Daily proportion of time feeding ranged from 0.017 to 0.85 for Greenland collars, 0.017–0.85 for Greenland backpacks, and 0.018–0.94 for midcontinent geese.

**Fig. 2:**
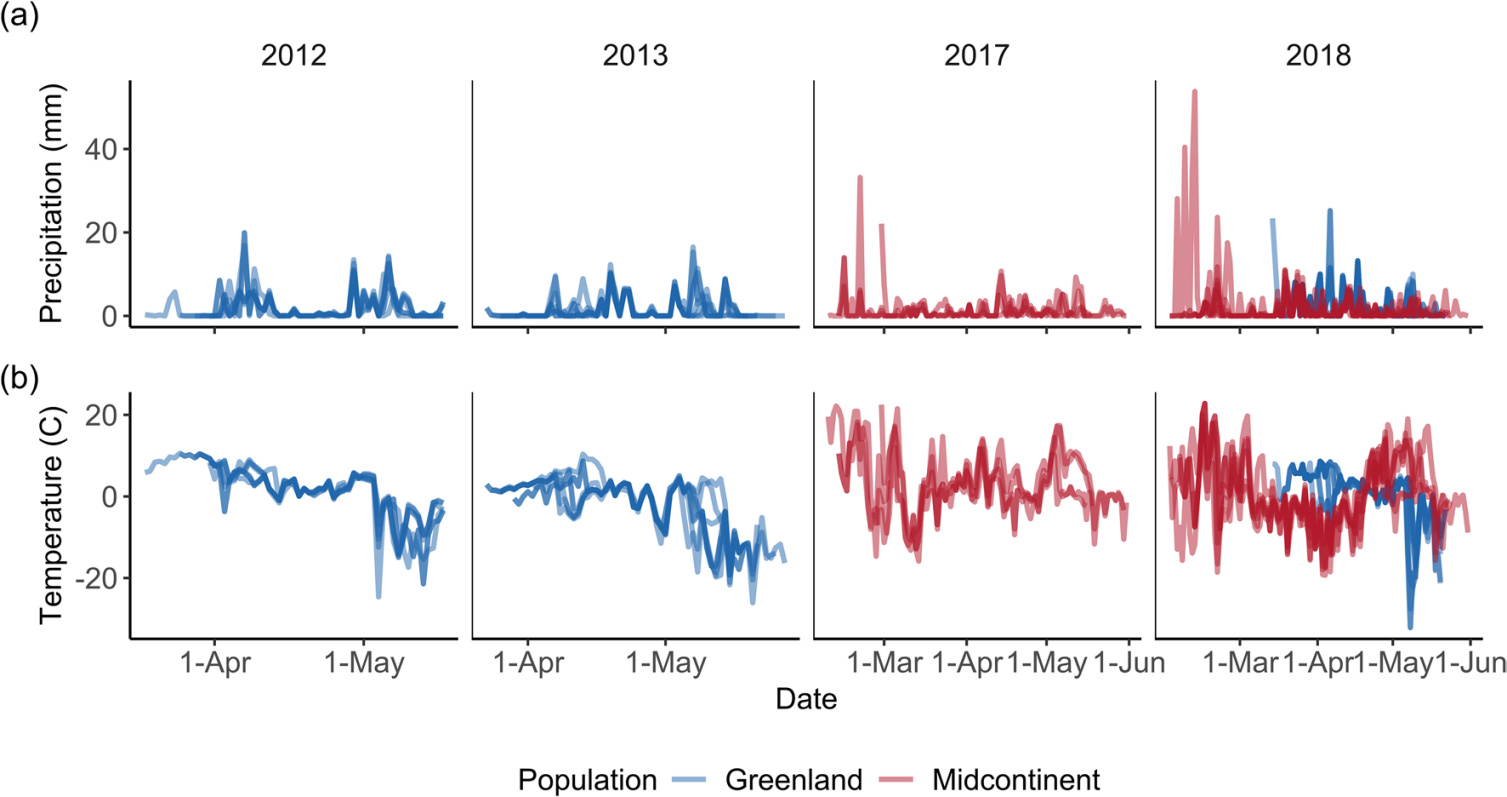
Time series of **a** precipitation and **b** minimum temperature encountered by 35 white-fronted geese in 2012–2013 (*n* = 15) and 2017–2018 (*n* = 20) in the midcontinent North American (red) and Greenland (blue) populations of white-fronted geese. Lines reflect conditions experienced by individual geese throughout migration, and darker lines indicate similar conditions between individuals

Minimum temperature appeared to be more variable between years for midcontinent geese (Fig. 2). The average minimum temperature for Greenland geese was overall slightly lower than midcontinent geese (− 0.1 C vs. 1.4 C; range − 32.2 to 10.6 vs. − 19.3 to 22.8), and Greenland geese experienced slightly more precipitation on average (1.9 mm vs. 1.3 mm), though individual events may have been greater for midcontinent geese than Greenland geese (53 mm vs. 25.2 mm maximum).

### Quantifying weather effects on PTF and ODBA

**Fig. 3:**
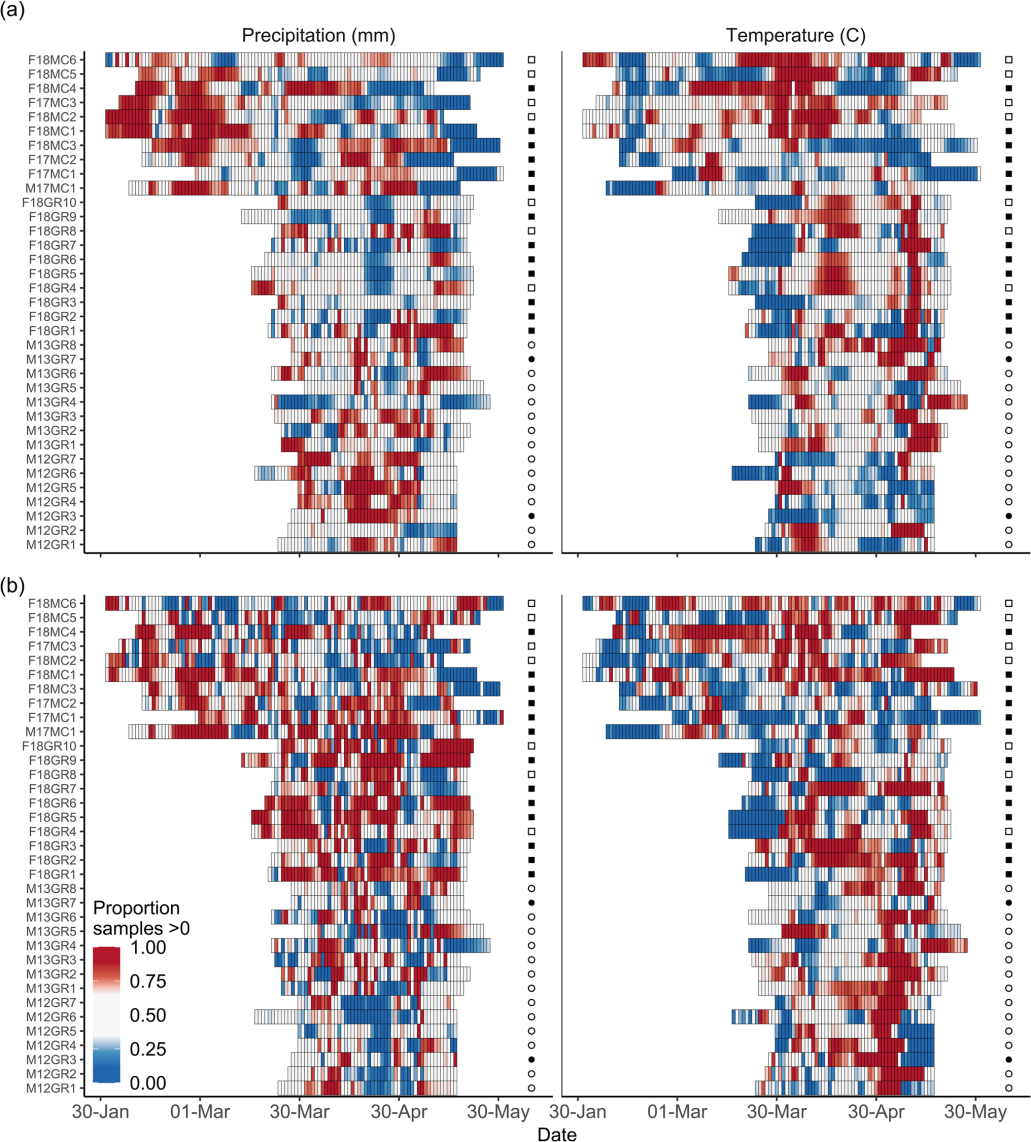
Daily effects of precipitation (mm) and minimum temperature (°C) on **a** energy expenditure and **b** proportion of time feeding for white-fronted geese. Each cell represents the posterior distribution of time-varying regression coefficients estimated via a random walk autoregressive process. Proportion of posterior samples greater or less than 0 is used to indicate strength of effect (e.g., stronger positive effect shown in red and stronger negative effect shown in blue). Individual identification codes on the y-axis include sex, year tagged, and population of each goose. Symbols on the right side of each plot indicate reproductive outcome; filled symbols represented reproductive success (geese tagged in 2012 or 2013) and attempted reproduction (geese tagged in 2017 or 2018), while open symbols indicate failure or deferral

Time-varying coefficients of the weather variables appeared to have stronger effects on PTF than lnODBA as there are more periods of time where the proportion of samples for the coefficients are close to 1 or 0 (Fig. 3), indicating greater certainty in the positive or negative effect. A majority of midcontinent geese showed a period of strong positive effects (i.e., > 80% posterior samples greater than 0) of precipitation on both response variables, PTF and lnODBA, at the beginning of migration, and in a positive effect of minimum temperature on lnODBA during March and April. Many Greenland geese showed a pattern of high-certainty positive effects of minimum temperature in both PTF and lnODBA in early May similar to midcontinent birds. Greenland geese did not show a consistent pattern in the effects of precipitation on lnODBA or PTF, though there was a small pattern of negative effects of precipitation on PTF in Greenland birds in 2012 that was not present in 2013 or 2018. The effects on PTF appear to be overall stronger than the effects on lnODBA.

### Antecedent effect of PTF and ODBA on probability of deferral

**Fig. 4:**
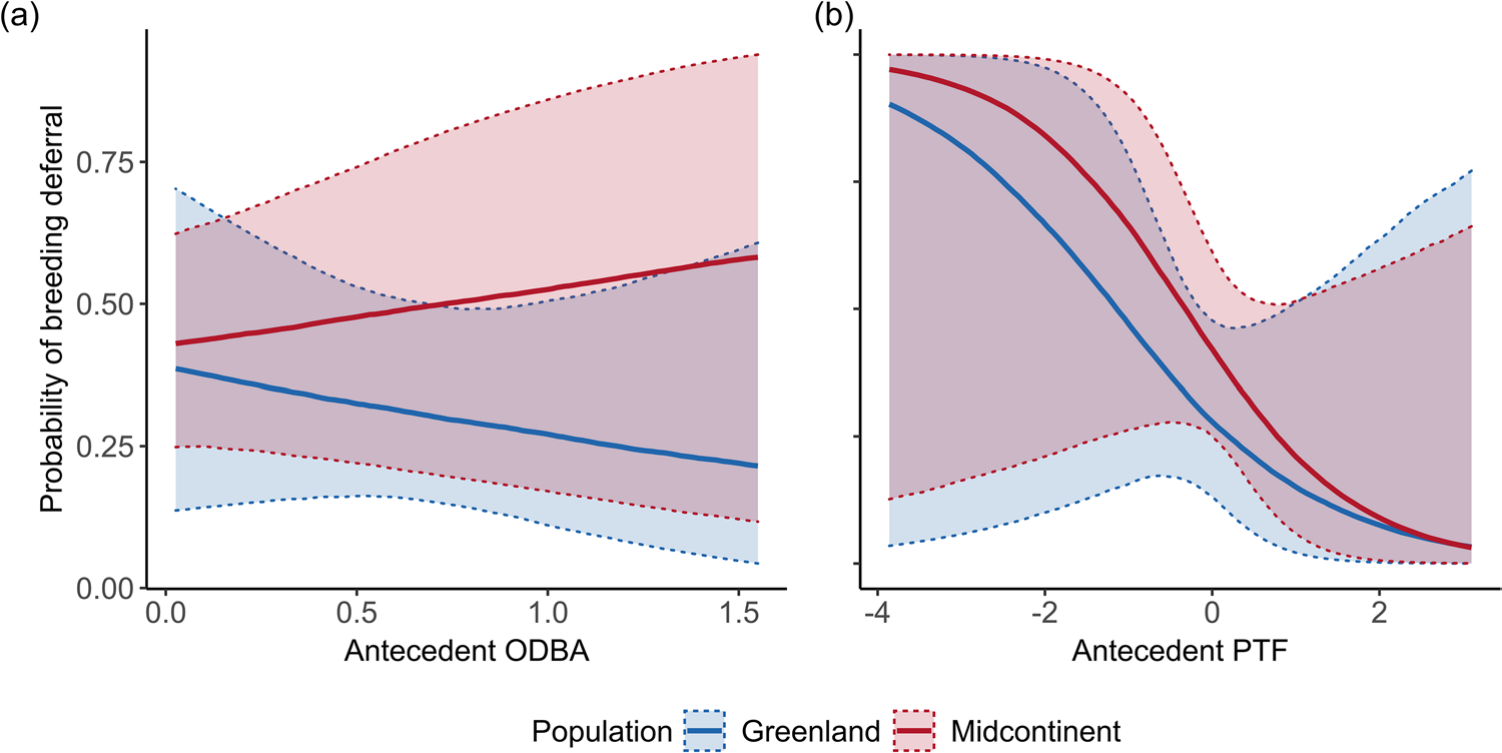
Predicted probability of deferring reproduction in Greenland (blue) and North American midcontinent (red) populations of greater white-fronted geese explained by **a** antecedent energy expenditure and **b** antecedent proportion of time feeding. Shading represents 95% credible intervals. X-axis values have been scaled and centered

The interaction of population and antecedent lnODBA (the sum of daily lnODBA multiplied by daily weight), but not antecedent PTF, explained moderate variation in probability of breeding deferral (Fig. 4, Supplementary File 1: Table S2). The odds of deferring reproduction in midcontinent geese decreased by a factor of 0.84 for each standard deviation increase in lnODBA, and increased by a factor of 0.32 for Greenland geese. The odds of deferring reproduction in midcontinent geese decreased by a factor of 0.72 with each standard deviation increase in PTF, and the odds of deferring decreased by a factor of 1.2 in Greenland geese. The probability of deferral for midcontinent geese with average PTF was 41.9% (95% CRI: 17.7%, 69.7%), while the probability of deferral for Greenland geese with average PTF was 27.9% (95% CRI: 6.4%, 67.0%; Fig. 4b). The models did not detect any differentially important time points for antecedent lnODBA or PTF, and all daily weights for both models were close to 0.02, which is equal to 1 divided by 54, the total number of days in the model (Supplementary File 1: Figure S2).

## Discussion

We used a combination of GPS and ACC data to assess daily relationships and the cumulative effect of animal decision-making on subsequent productivity, measured as the probability of breeding deferral. Many Greenland geese spent more time foraging when the day was warmer, approximately the last 1–2 weeks prior to leaving Iceland (Fig. 3). This pattern was most prominent in male geese from 2012 to 2013, though there did not appear to be differences in birds that successfully reproduced and those that did not. Temperature was expected to be linked to forage quality in spring (van Wijk et al. 2012), so this positive association could be a result of increased plant growth providing greater opportunities for geese to feed prior to migration from Iceland to Greenland. Previous work has shown that increased temperatures earlier in the year are positively correlated with increased accumulation of fat storage prior to departure from Ireland (Fox and Walsh 2012).

Similarly, midcontinent geese showed a positive association of energy expenditure and, to an extent, time foraging with precipitation in the early weeks of the spring migration period. The increased precipitation could be linked to ideal growing seasons of crops such as winter wheat (Stone and Schlegel 2006) that we speculate the geese could have been exploiting. While there appears to be variation between individuals in the effects of time-varying coefficients, we did not observe patterns that consistently varied across reproductive status. However, light-bellied brent geese (*Branta bernicla hrota*) follow an almost identical migratory route to Greenland white-fronted geese, but migrate further west to North America, with documented effects of weather conditions on reproductive success (Harrison et al. 2013; Cleasby et al. 2017). Thus, further investigation into a broader suite of weather conditions may reveal different patterns in daily and cumulative effects on energy expenditure and reproductive success. We reiterate that our study captured only a small portion of potential conditions, as circumstances can vary greatly among years (e.g., Dickey et al. 2008).

Habitats used by individuals of the two populations during spring stopovers can be quite different, and we expected that this would be reflected in differences in ODBA and PTF between populations. Greenland geese feed primarily on grass in hay meadows and limited waste grain in Iceland (Boyd et al. 1998; Fox and Walsh 2012), while midcontinent geese feed extensively on waste grain (Krapu et al. 1995; Ely et al. 2013). Geese tend to feed longer on grasses than agricultural grains, and while most grasses are higher in protein, they are lower in lipid content compared to agricultural grains (Ely and Raveling 2011). However, we found that greater overall time foraging during spring migration was associated with a lower probability of breeding deferral in both populations, highlighting the need for future research about habitat use throughout migration and nutrient quality of foods consumed by white-fronted geese in Europe and North America.

Our results indicated that the strength and direction of the relationship between antecedent time spent foraging and probability of deferral was similar between populations, but the strength of relationships between probability of deferral and energy expenditure were different between populations. As predicted, more time foraging lowered the probability of breeding deferral. This likely indicates that more time spent foraging is related to greater resource reserves for breeding once geese arrive in the Arctic. We hypothesize that the negative relationship in probability of deferral and energy expenditure could be from geese meeting their energetic requirements and loafing in Iceland until moving to Greenland (Fox et al. 2012), and any birds that are still moving and foraging heavily have likely not acquired sufficient resources to breed. The increase in energy expenditure associated with higher deferral rates in midcontinent geese could be a result of disturbance preventing geese from obtaining necessary resources due to increased flight during stopovers (Béchet et al. 2004). Alternatively, the increased energy expenditure could be a result of difference in space use at stopovers, as other species show substantial individual variation in flight distance to foraging sites (Clausen et al. 2013). Midcontinent geese have been observed moving approximately 20 km per day between roost and feeding sites (Pearse et al. 2013). Consistently longer flight distances from roost sites to foraging areas could indicate poor decision making in individuals that could lead to subsequent reproductive failure due to a negative energy balance.

Our analyses did not identify any important time periods in which birds that deferred reproduction differed from those who attempted (or those that were successful *vs* failed in 2012–2013). While we emphasize the limitations in our ability to discern early failure (i.e., ≤ 3 days) from deferred breeding, which may be the result of different mechanisms, we observed breeding deferral in fewer than half of the geese in both populations, which was lower than expected for the Greenland geese given the declining population, though out of the males fitted with backpack devices, only 2 out of 15 returned to wintering areas with offspring. Therefore, we interpret our results to indicate that despite parental preparation, young are not surviving to be recruited into the population by the time geese return to wintering areas. Goslings are vulnerable to a variety of predators (Anthony et al. 1991; Bowman et al. 2004) and harsh weather (Fondell et al. 2008). Additionally, warming climates and early onset of spring can lead to increased mortality of offspring due to a mismatch between gosling growth and peak food quality, regardless of parent abilities to refuel after arriving in the Arctic (Lameris et al. 2018). Given the mismatch between the number of geese attempting reproduction and geese returning to wintering areas with offspring, we suggest investigation into rates of nest failure and brood loss in Greenland white-fronted geese to explain differences in productivity between these two populations.

Our study provides an initial example of blending temporally frequent ACC data with GPS data for birds of contrasting migration routes to uniquely quantify how individuals respond to their environment and the implications of individual behavioral patterns on reproduction. Inferences from tracking studies are commonly limited because of the relatively low number of individuals tagged. Yet, advances in miniaturized tracking technologies such as accelerometers allows for a substantial amount of information to be collected from each individual. The method demonstrated in this paper may increase our capacity to link animal behavior and individual reproductive output, as new methods are being derived for identifying reproductive events remotely (e.g., Schreven et al. 2021; Ozsanlav-Harris et al. 2022), with environmental conditions (Valletta et al. 2017) and consequences of climate change (e.g., Lameris et al. 2018). Further, these methods could be used in the context of varying landscapes to better understand linkages among behavior, environmental conditions, and reproductive success, allowing practitioners to pinpoint critical periods of the annual cycle to ascribe priority areas for improved conservation efforts.

## Supplementary Information

Below is the link to the electronic supplementary material.

Supplementary file 1 (DOCX 310 KB)

## Data Availability

The data analyzed in the current study are available from the corresponding author upon reasonable request.

## Code availability

Code for analyses is available on GitHub (github.com/s-cunningham/GeeseBehavior-Weather).
