# Supplementary material for: Time-varying effects of local weather on behavior and probability of breeding deferral in two Arctic-nesting goose populations
Source: Oecologia. 2022 Dec 28;201(2):369–83. doi: 10.1007/s00442-022-05300-x (PMC9944342; doi:10.1007/s00442-022-05300-x)
Supplement: Supplementary file 1 — (DOCX 310 KB) [file 442_2022_5300_MOESM1_ESM.docx]

**SUPPORTING INFORMATION**

**Time-varying effects of weather on behavior and probability of breeding deferral in two Arctic-nesting goose populations**

Stephanie A. Cunningham, Toryn L. J. Schafer, Christopher K. Wikle, Jay A. VonBank, Bart M. Ballard, Lei Cao, Stuart Bearhop, Anthony D. Fox, Geoff M. Hilton, Alyn J. Walsh, Larry R. Griffin, Mitch D. Weegman

Model code is available on GitHub: <https://github.com/s-cunningham/GeeseBehavior-Weather>

**Table S1.** Tag- and behavior specific classification success for Ornitela and CTT transmitters for three behaviors classified with a random forest. Performance metrics based on 10-fold cross-validation.

|  | **Ornitela (93.1% overall accuracy)** | | | **CTT (97.8% overall accuracy)** | | |
| --- | --- | --- | --- | --- | --- | --- |
|  | **Fly** | **Graze** | **Stationary** | **Fly** | **Graze** | **Stationary** |
| Precision | 92.7 | 89.1 | 100.0 | 100.0 | 100.00 | 94.1 |
| Recall | 86.4 | 95.0 | 97.6 | 100.0 | 93.7 | 100.0 |
| Accuracy | 93.8 | 93.1 | 99.3 | 100.0 | 97.8 | 97.8 |

**Table S2.** Regression coefficients from stochastic antecedent models, where mean is the parameter estimate,

| ***Model***  **Parameter** | **Mean** | **SD** | **95% CRI** | **Proportion samples > \| < 0** |
| --- | --- | --- | --- | --- |
| *lnODBA* |  |  |  |  |
| Intercept | -0.30 | 0.63 | -1.55, 0.89 | 0.68 |
| Antecedent lnODBA | 0.41 | 1.02 | -1.60, 2.40 | 0.65 |
| Flyway | -0.18 | 1.17 | -2.46, 2.12 | 0.56 |
| Flyway * lnODBA interaction | -0.95 | 1.28 | -3.43, 1.62 | 0.77 |
| *PTF* |  |  |  |  |
| Intercept | -0.33 | 0.60 | -1.54, 0.83 | 0.71 |
| Antecedent PTF | -1.01 | 1.08 | -3.08, 1.08 | 0.83 |
| Flyway | -0.65 | 0.87 | -2.36, 1.03 | 0.78 |
| Flyway * PTF interaction | 0.17 | 1.21 | -2.22, 2.56 | 0.56 |


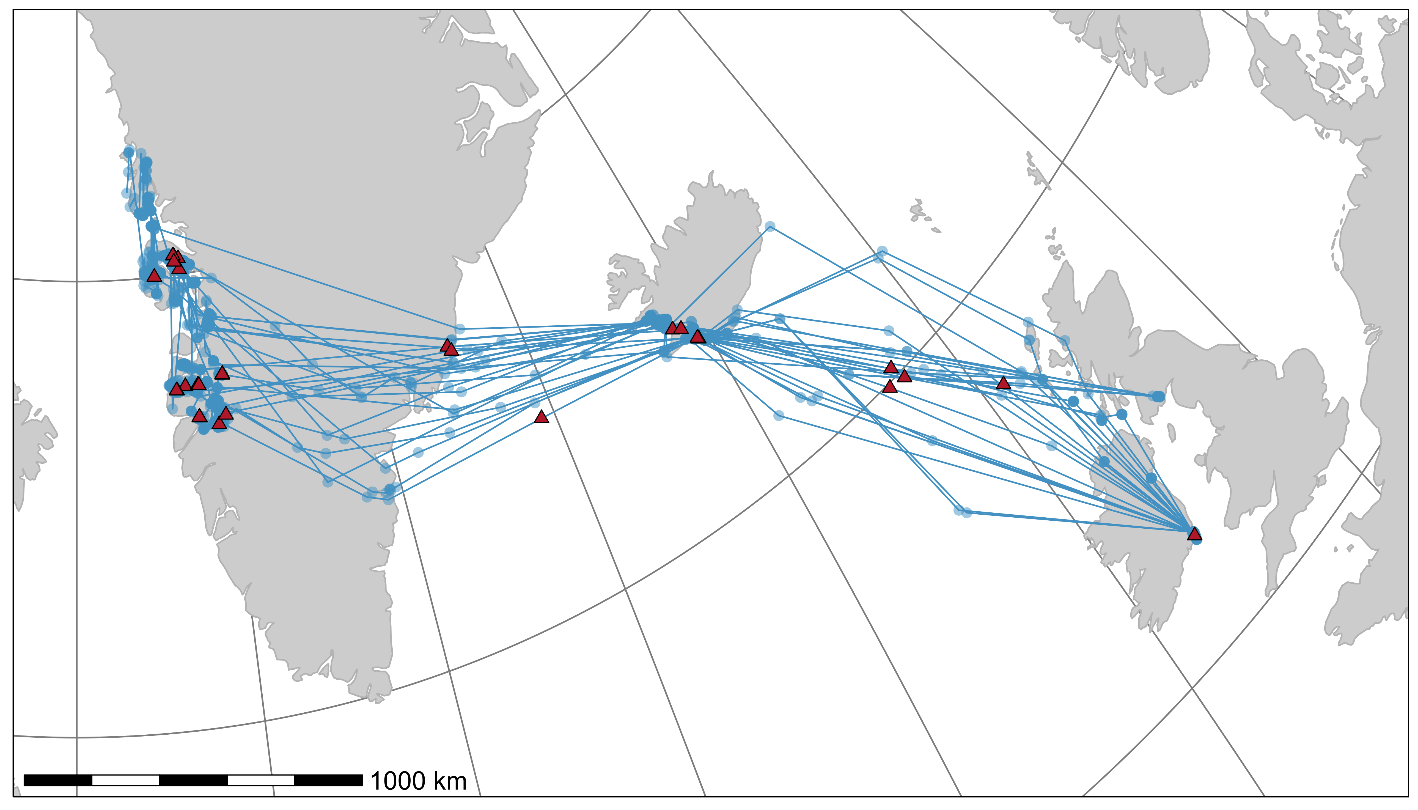


**Figure S1.** Daily GPS locations of male greater white-fronted geese with backpack e-obs devices in 2012 and 2013 with interpolated points in red triangles.


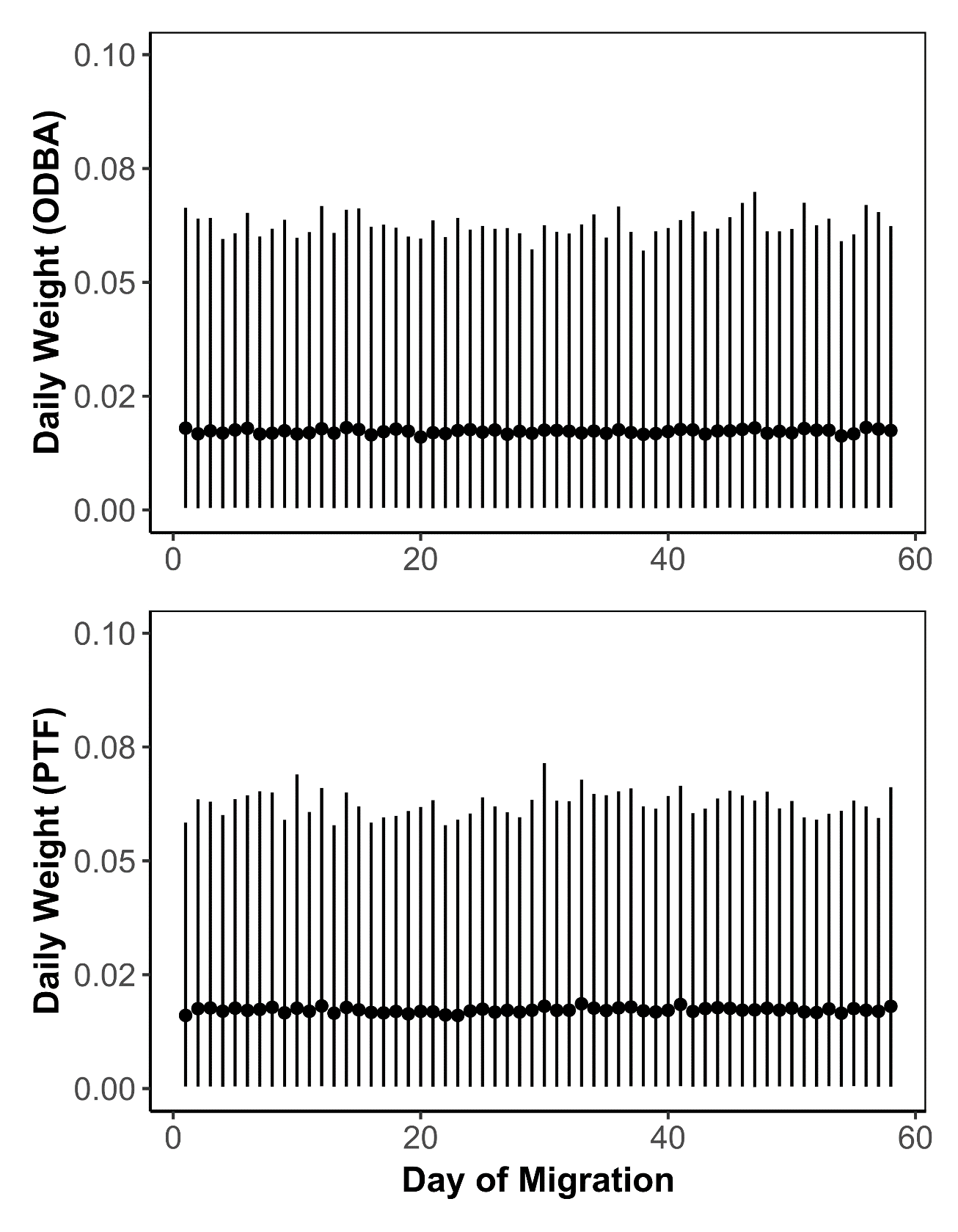


**Figure S2.** Daily weights for each combination of population and antecedent variable (overall dynamic body acceleration [ODBA] and proportion of time spent feeding [PTF]). Points indicate posterior, with bars showing posterior 95% credible interval.
